# Minimally Invasive Surgery for Mitral Valve Endocarditis: A Systematic Review and Meta-Analysis of Reconstructed Time-to-Event Data

**DOI:** 10.3390/jpm16070350

**Published:** 2026-06-29

**Authors:** Thomas Karagkounis, Angeliki Alifragki, Ioannis Zoupas, Sofia Sarantou, Nikolaos Schizas, Konstantinos S. Mylonas, Dimitrios C. Iliopoulos

**Affiliations:** 1 4th Department of Cardiac Surgery, Hygeia Hospital, 15123 Athens, Greece; 2 Medical School, University of Crete, 70013 Heraklion, Greece; 3 Department of Cardiothoracic and Vascular Surgery, McGovern Medical School, The University of Texas Health Science Center at Houston, Houston, TX 77030, USA; 4 Department of Surgical Oncology, The University of Texas MD Anderson Cancer Center, Houston, TX 77030, USA; 5 Division of Cardiothoracic Surgery, Emory University School of Medicine, Atlanta, GA 30322, USA; 6 Laboratory of Experimental Surgery and Surgical Research ‘N.S. Christeas’, National and Kapodistrian University of Athens, 11527 Athens, Greece

**Keywords:** minimally invasive surgery, infective endocarditis, mitral, overall survival

## Abstract

**Background/Objectives**: Minimally invasive (MIS) mitral valve surgery has been proven to be a safe and effective alternative to median sternotomy (ST), with advantages in postoperative recovery and morbidity. However, its role in the setting of infective endocarditis (IE) remains uncertain. This meta-analysis aims to evaluate the outcomes of MIS in mitral valve surgery for infective endocarditis. **Methods**: A PRISMA-compliant search for studies including patients undergoing MIS for mitral valve IE was performed through 14 January 2026, in PubMed, Scopus and Cochrane. Time-to-event data were reconstructed from published Kaplan–Meier curves. A secondary comparative analysis focusing on MIS versus ST techniques was conducted. **Results**: Fourteen retrospective studies comprising 949 patients were analyzed. In the MIS cohort, early mortality was 4.2% (95%CI: 1.8%, 7.4%). Overall survival was 86.7% at 1 year, 75.2% at 5 years and 56.2% at 10 years. Freedom from IE-related reoperation remained high at 97.5%, 95.9%, and 90.7% at 1, 5, and 10 years, respectively. Mitral valve repair was performed in 52.5% of patients. In secondary comparative analyses, overall survival at 4-year follow-up was not different between MIS and ST [HR: 0.82 (95%CI: 0.43, 1.57), *p* = 0.55]. MIS was associated with a significantly shorter intensive care unit (ICU) stay [MD: −1.52 days (95%CI: −2.08, −0.97), *p* < 0.01]. **Conclusions**: MIS for mitral valve IE is associated with favorable early and long-term outcomes, comparable survival with sternotomy, and reduced ICU stay. These findings suggest that MIS may be considered as a feasible and potentially effective alternative for the management of mitral valve IE in carefully selected patients. Further prospective comparative studies are warranted.

## 1. Introduction

Infective endocarditis (IE) remains a life-threatening condition, with reported in-hospital mortality rates ranging from 17% to 33% [1,2]. Nearly half of patients with left-sided disease ultimately require surgical treatment and mitral valve involvement occurs in approximately 40% of cases [3,4,5]. Expedited mitral surgery is indicated in the presence of heart failure due to valvular dysfunction, infection caused by highly resistant organisms, uncontrolled or persistent infection, and prosthetic valve involvement [6]. Given the complexity of mitral IE, current guidelines from the American Association for Thoracic Surgery favor a traditional median sternotomy, which provides optimal exposure and facilitates management of advanced disease and unexpected intraoperative findings [7,8].

Minimally invasive mitral valve surgery has emerged as a safe and effective alternative to conventional approaches, offering faster postoperative recovery, shorter hospital stay, improved cosmetic outcomes, and excellent long-term survival [9,10,11,12,13,14]. The most commonly employed technique is right mini-thoracotomy, typically performed through a 4–7 cm lateral incision in the fourth or fifth intercostal space, with either video-assisted or fully endoscopic visualization [10,14]. Variations in this technique include the transaxillary approach, which places the incision within the axillary fold to enhance cosmetic results [11]. Robotic-assisted surgery, in contrast, utilizes multi-articulated instruments and high-definition visualization to facilitate complex repairs through small ports in the right hemithorax [12].

Despite its increasing adoption, evidence regarding postoperative and long-term outcomes of minimally invasive surgery (MIS) in the specific context of mitral valve infective endocarditis (MVIE) remains limited, particularly in direct comparison with traditional surgical techniques. To fill this gap in the literature, we performed a systematic review and meta-analysis evaluating the pooled outcomes of MIS for MVIE and comparing these techniques with traditional sternotomy. To improve precision, we reconstructed time-to-event outcomes from the original Kaplan–Meier curves.

## 2. Materials and Methods

### 2.1. Systematic Research and Eligibility Criteria

The study adhered to the Preferred Reporting Items for Systematic Reviews and Meta-Analyses (PRISMA) statement and was performed according to a prespecified protocol agreed upon by all authors [15]. Eligible studies were published in English and reported original patient data, including clinical studies, cohort studies, and case series. We included studies enrolling patients with mitral valve endocarditis undergoing minimally invasive surgery, with or without a comparator group treated with conventional techniques. Reviews, meta-analyses, case reports, studies not relevant to the research question, and non-English publications were excluded. The PICO criteria (Population, Intervention, Comparison and Outcome) were utilized to shape our research question:

1. Population: Patients with mitral valve infective endocarditis (MVIE)
2. Intervention: Patients who underwent minimally invasive surgery (MIS)
3. Comparison: (a) none for the primary single-arm meta-analysis and (b) conventional full sternotomy for the secondary comparative meta-analysis
4. Outcomes: The primary endpoints for the primary single-arm meta-analysis were overall survival (OS) and freedom from infective endocarditis (IE) related reoperation, and secondary endpoints included demographics, intraoperative and postoperative characteristics, and complication rates. For the secondary comparative meta-analysis, primary outcomes were the hazard ratio (HR) for long-term survival and duration of hospital and intensive care unit stay, with secondary outcomes defined as above.

Relevant studies were identified through a systematic search of the PubMed/MEDLINE, EMBASE, and Cochrane databases up to 14 January 2026. The databases were searched independently by two reviewers (TK and AA) using the following search strategy: (minimally OR ministernotomy OR thoracotomy OR minithoracotomy OR hemisternotomy OR thoracoscopic OR endoscopic OR robotic) AND (endocarditis OR ie) AND (mitral OR left OR atrioventricular). All conflicts regarding study eligibility were resolved with the assistance of an additional reviewer (IZ). Rayyan reference management software was used throughout all stages of the literature search and study selection process [16]. The PRISMA checklist (Table S5) is included in the Supplementary Materials. The study protocol was prospectively registered in PROSPERO (ID number: CRD420261370652).

### 2.2. Data Extraction

Data extraction was conducted independently by two reviewers (TK and AA) using a standardized, predefined data extraction form. Potential conflicts were resolved after discussion with another author (IZ). The following variables were collected for the MIS cohort and, when available, for the ST cohort: age, sex, body mass index, history of prior cardiothoracic surgery, chronic obstructive pulmonary disease, hypertension, diabetes mellitus, dyslipidemia, prior myocardial infarction, heart failure, peripheral vascular disease, cerebrovascular accident, chronic kidney disease, presence of shock presentation, dialysis, left ventricular ejection fraction and presence of active infective endocarditis (IE). Mitral valve-specific variables were extracted for both groups, including valve type (native or prosthetic), presence of regurgitation and prolapse, vegetations, abscess formation, and leaflet perforation. Microbiological data, including the causative pathogen, were also collected.

Intraoperative variables included total operative time, cardiopulmonary bypass time, aortic cross-clamp time and cross-clamping strategy as well as fibrillatory arrest information when applicable. Data on surgical management of the mitral valve -repair versus replacement- as well as the type of prosthesis implanted (mechanical or biological) were recorded. Concomitant procedures and conversion to full sternotomy were also documented.

Postoperative variables included length of hospital stay, intensive care unit stay, duration of mechanical ventilation, and postoperative complications, including arrhythmia, stroke, multi-organ failure, reintubation and sepsis. The need for permanent pacemaker implantation, extracorporeal membrane oxygenation support, red blood cell transfusion, reoperation for bleeding and mitral valve sufficiency at discharge were also recorded. Early mortality was defined as 30-day postoperative mortality.

### 2.3. Statistical Analysis

#### 2.3.1. Data Pooling and Patient Feature Meta-Analysis

The techniques of Hozo et al. and Wan et al. were utilized to calculate the standard deviations (SDs) and mean values of continuous variables whenever medians and ranges or median and interquartile ranges were provided [17,18].

In the primary single-arm meta-analysis, relative rates and 95% confidence intervals (CI) were calculated for categorical data. When data were available in fewer than three studies, only descriptive statistical analysis was performed.

For the secondary, comparative meta-analysis, categorical outcomes were summarized using odds ratios (OR) with 95% CI, whereas continuous variables were analyzed using mean differences (MD). Statistical significance was defined as a two-sided *p*-value of less than 0.05. Random effects models (DerSimonian–Laird) were adopted. Forest plots were generated to display results. To assess the occurrence rates of several events and the presence of between-study heterogeneity, we used the χ^2^-based Q statistic (significant if *p* < 0.1) and I2. All analyses were performed using STATA BE 19.5 (StataCorp LLC, College Station, TX, USA).

#### 2.3.2. Reconstruction of Patient Time-to-Event Data and Survival Meta-Analysis

Where available, individual patient data (IPD) were reconstructed from Kaplan–Meier (KM) curves of included studies using the method described by Wei and Royston [19]. High-resolution KM curves were digitized using Web Plot Digitizer (version 5) [20]. Extracted data points were processed such that each point represented a time-to-event observation derived from survival probabilities. Monotonicity violations were corrected using a pool–adjacent–violators algorithm. Reconstructed IPD were subsequently analyzed in Stata BE 19.5 (StataCorp, College Station, TX, USA) to produce pooled Kaplan–Meier curves across studies, enabling estimation of aggregated survival outcomes. When KM curves were not available in a study but information on follow-up time and event status was reported, survival data were imported into Stata BE 19.5 and KM curves were reconstructed for the respective study. The original and reconstructed KM curves are provided in the Supplementary Materials. If risk tables and/or censoring information were available, these data were incorporated into the reconstructed Kaplan–Meier curves (Supplementary Figures S1–S11).

#### 2.3.3. Individual Patient Data Survival Meta-Analysis

We utilized the KM method to measure overall survival (OS) and freedom from IE-related reoperation for the MIS cohort.

To compare OS differences between the MIS and ST groups, in our secondary meta-analysis, we used the Cox proportional hazards regression model. In this model, each patient within a given study is assumed to have a similar risk of experiencing an event as other patients in the same study. For these Cox models, the proportional hazards assumption was verified by comparing the predicted and observed survival. Reconstructed curves were generated using the KM product limit method and the Hazard Ratios (HR) and 95%CIs were calculated for each group.

#### 2.3.4. Publication Bias Assessment and Meta-Regression for the Primary Meta-Analysis

In the primary meta-analysis, publication bias was evaluated via funnel plot and Egger’s test for the single-arm proportions that were available in ten or more studies.

A random-effects meta-regression was performed to investigate heterogeneity in the single-arm proportions that were available for ten or more studies and to evaluate the impact of study publication year and proportion of robotic-assisted surgery. A *p*-value < 0.05 was considered statistically significant.

### 2.4. Risk-of-Bias Assessment

Quality assessment of included case series was performed using the National Heart, Lung, and Blood Institute (NHLBI) scale. Risk of bias in comparative studies was evaluated using the Risk of Bias in Non-randomized Studies of Interventions (ROBINS-I) tool, which categorizes studies as low, moderate, serious, or critical risk of bias. A follow-up duration of at least 12 months after the index operation was considered sufficient. Assessments were independently conducted by two reviewers (TK and SS), with disagreements resolved by consensus in consultation with an experienced reviewer (IZ). Mean quality scores and standard deviations were calculated across all included studies.

### 2.5. Certainty of Evidence Assessment

The certainty of evidence for the primary and secondary outcomes was assessed using the Grading of Recommendations Assessment, Development and Evaluation (GRADE) approach for comparative observational studies [21,22]. Observational studies initially started at low certainty of evidence. The certainty was downgraded based on five domains: risk of bias (informed by the ROBINS-I), inconsistency (unexplained heterogeneity between studies), indirectness, imprecision (wide confidence intervals), and publication bias. The certainty of evidence could be upgraded in the presence of a large magnitude of effect (odds ratio > 5). The final certainty of evidence for each outcome was categorized as high, moderate, low, or very low.

## 3. Results

### 3.1. Study Selection

**Figure 1:**
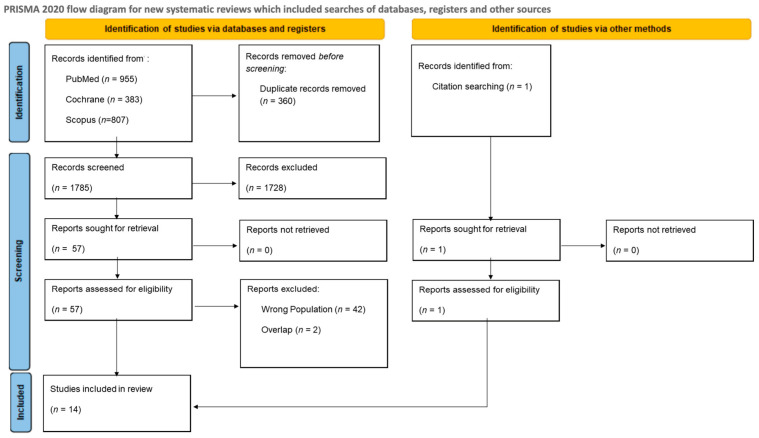
PRISMA flow-diagram.

The initial literature search yielded 2145 potentially eligible articles. After removal of duplicate records and unrelated papers, fifty-seven (57) studies were fully reviewed for eligibility. Ultimately, after the removal of overlapping studies [23,24], fourteen retrospective studies were included [25,26,27,28,29,30,31,32,33,34,35,36,37,38]. Four comparative studies evaluating minimally invasive surgery (MIS) versus full sternotomy (ST) and contributing to the primary single-arm MIS analysis as well [25,26,30,34], while ten studies contributed only to the primary single-arm MIS analysis [27,28,29,31,32,33,35,36,37,38] (Figure 1).

In total, data from 949 patients who underwent surgical management for mitral valve infective endocarditis (MVIE) were analyzed (Table 1). There was no overlap among eligible studies with regard to included patients, recruitment periods or centers.

### 3.2. Primary Analysis—Pooled Outcomes for the MIS Group

#### 3.2.1. Basic Demographics and Past Medical History—MIS

In the MIS cohort, the mean age was 59.2 ± 14.5 years and 62.8% (95%CI: 57.9%, 67.5%) of patients were male. The mean body mass index (BMI) was 25.8 ± 6.1 kg/m^2^. Chronic obstructive pulmonary disease was present in 13.5% (95%CI: 5.0%, 24.8%) of patients. Hypertension (HTN) was reported in 55.9% (95%CI: 46.8%, 64.8%), diabetes mellitus (DM) in 20.3% (95%CI: 10.8%, 31.7%), and heart failure in 20.6% (95%CI: 2.3%, 48.2%). Peripheral vascular disease was present in 2.9% (95%CI: 1.0%, 5.6%), prior cerebrovascular events in 23.8% (95%CI: 9.5%, 41.9%), chronic kidney disease in 15.7% (95%CI: 10.6%, 21.6%), and dialysis dependence in 5.6% (95%CI: 2.5%, 9.5%). Prior myocardial infarction was reported in 6.2% (95%CI: 2.3%, 13.0%) and prior cardiothoracic surgery in 14.8% (95%CI: 8.7%, 22.0%).

The mean preoperative left ventricular ejection fraction (LVEF) was 59.6 ± 8.6%. Active infective endocarditis was present in 81.6% (95%CI: 50.4%, 99.6%) of patients, and 10.0% (95%CI: 3.3%, 21.8%) presented with shock at admission. Baseline characteristics for the MIS cohort are summarized in Supplementary Table S1.

#### 3.2.2. Preoperative Characteristics of the Diseased Mitral Valve (MV)—MIS

In the MIS cohort, the affected valve was native in 93.0% (95%CI: 77.6%, 100%) and prosthetic in 7.0% (95%CI: 0.0%, 22.4%). MV prolapse was present in 45.9% (95%CI: 35.0%, 57.0%) of patients, while mitral regurgitation was reported in 49.7% (95%CI: 0.0%, 100%). Vegetations were identified in 91.8% (95%CI: 67.6%, 100%), abscess formation in 7.0% (95%CI: 2.9%, 12.5%) and leaflet perforation in 19.1% (95%CI: 8.0%, 33.1%) of MIS patients. The anterior leaflet was involved in 50.4% (95%CI: 15.1%, 85.5%) of cases.

Microbiological analysis demonstrated staphylococcal species in 29.7% (95%CI: 24.3%, 35.5%) of cases, of which 84.3% (95%CI: 71.4%, 93.0%) were Staphylococcus aureus. Streptococcal species were identified in 23.4% (95%CI: 18.5%, 28.9%) and enterococcal species in 10.2% (95%CI: 6.9%, 14.5%) of cases. Other pathogens accounted for 6.6% (95%CI: 3.9%, 10.2%), while culture-negative endocarditis was reported in 9.9% (95%CI: 6.6%, 14.1%). Microbiological data were not available in 20.1% (95%CI: 15.5%, 24.4%) of cases. Diseased MV characteristics are summarized in Table 2.

#### 3.2.3. Intraoperative Details—MIS

The mean total operative time was 195.0 ± 61.4 min, with a cardiopulmonary bypass (CPB) time of 130.6 ± 38.7 min and an aortic cross-clamp time of 92.0 ± 29.1 min. Aortic cross-clamp was used in 82.3% (95%CI: 31.2%, 100%), endo-aortic balloon occlusion in 17.7% (95%CI: 0.0%, 68.5%) and fibrillatory arrest in 0.1% (95%CI: 0.0%, 0.6%) of cases. Robotic-assisted surgery was performed in 5.4% (95%CI: 0.0%, 18.4%) of MIS patients, while the remaining patients underwent totally endoscopic techniques, right mini-thoracotomy with video assistance, or direct vision approaches.

Mitral valve repair was performed in 52.5% (95%CI: 38.1%, 66.6%) of patients, with 72.0% (95%CI: 57.5%, 83.8%) of these involving mitral annuloplasty, whereas mitral valve replacement was undertaken in 47.5% (95%CI: 33.4%, 61.8%). Among those undergoing valve replacement, mechanical prostheses were implanted in 34.6% (95%CI: 27.1%, 42.6%) and bioprosthetic valves in 65.4% (95%CI: 57.4%, 72.8%).

Concomitant tricuspid valve (TV) procedures were performed in 4.1% (95%CI: 1.4%, 7.8%) of cases and atrial septal defect closure in 5.4% (95%CI: 2.6%, 9.8%). Conversion to full sternotomy occurred in 2.3% (95%CI: 0.6%, 5.5%). Intraoperative details are summarized in Table 3.

#### 3.2.4. Postoperative Outcomes—MIS

The mean duration of mechanical ventilation was 11.6 ± 8.6 h, the mean intensive care unit stay was 1.8 ± 1.7 days, and the mean hospital length of stay was 8.8 ± 7.6 days.

The most frequently reported postoperative complication was arrhythmia, occurring in 13.1% (95%CI: 3.8%, 26.1%) of patients in the MIS group. Other complications included multiorgan failure in 6.3% (95%CI: 2.1%, 14.1%), acute kidney injury (AKI) in 4.7% (95%CI: 2.2%, 7.8%), sepsis in 1.4% (95%CI: 0.1%, 3.7%) and stroke in 1.4% (95%CI: 0.2%, 3.1%). Prolonged ventilation occurred in 12.3% (95%CI: 4.9%, 22.1%), reoperation for bleeding in 5.1% (95%CI: 0.1%, 14.3%), reintubation in 3.3% (95%CI: 1.1%, 9.1%), permanent pacemaker implantation in 2.8% (95%CI: 0.8%, 5.8%), and extracorporeal membrane oxygenation (ECMO) support in 1.8% (95%CI: 0.4%, 5.1%). Mean red blood cell transfusion was 1.7 ± 2.4 units.

At discharge, 87.6% (95%CI: 79.4%, 93.4%) of patients had no or trace mitral regurgitation, 11.3% (95%CI: 5.8%, 19.4%) had mild regurgitation and 1.1% (95%CI: 0.0%, 5.6%) had moderate regurgitation. Postoperative outcomes for the MIS cohort are visualized in Supplementary Table S2.

#### 3.2.5. Mortality and Overall Survival (OS)—MIS

**Figure 2:**
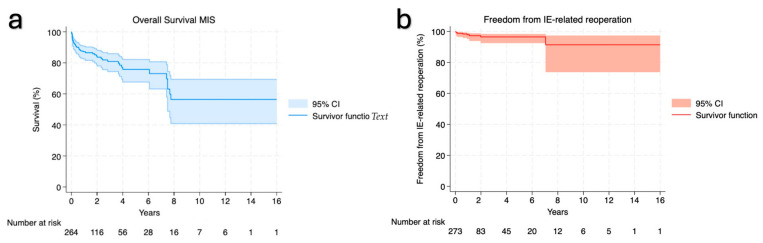
Reconstructed Kaplan–Meier curves for (**a**) overall survival and (**b**) freedom from infective endocarditis-related reoperation following minimally invasive surgery for mitral valve endocarditis.

Early (30-day) mortality in the MIS group was 4.2% (95%CI: 1.8%, 7.4%). Overall survival was 86.7% (95%CI: 81.9%, 90.2%) at 1 year, 75.2% (95%CI: 67.2%, 81.2%) at 5 years, and 56.2% (95%CI: 40.8%, 69.0%) at 10 years (Figure 2a). The KM curve for OS in patients undergoing MIS was reconstructed using survival data extracted from six studies [28,29,30,32,34,35].

#### 3.2.6. Reoperation and Freedom from IE-Related Reoperation—MIS

Early reoperation occurred in 6.0% (95%CI: 3.0%, 10.4%). Freedom from IE-related reoperation was 97.5% (95%CI: 94.8%, 98.6%) at 1 year, 95.9% (95%CI: 92.5%, 97.8%) at 5 years and 90.7% (95%CI: 74.8%, 97.4%) at 10 years (Figure 2b). The KM curve for freedom from IE-related reoperation in patients undergoing MIS was reconstructed using survival data extracted from five studies [26,28,29,30,32].

#### 3.2.7. Publication Bias Assessment and Meta-Regression—MIS

Funnel plot for the logit proportion of MV replacement revealed publication bias (Egger’s = 0.007) (Supplementary Figure S12).

A random-effects meta-regression was performed to evaluate potential associations between study-level variables and the logit proportion of MV replacement. The analysis did not reveal interference between the MV replacement proportion and the year of publication (*p* = 0.791) or the proportion of robotic-assisted surgery (*p* = 0.061) (Supplementary Figures S13 and S14).

### 3.3. Secondary Analysis—MIS Techniques vs. ST

For the secondary comparative meta-analysis, four comparative studies were included [25,26,30,34].

#### 3.3.1. Basic Demographics and Past Medical History—MIS vs. ST

Native valve infective endocarditis was significantly more frequent in the MIS group [OR: 3.10 (95%CI: 1.18, 8.15), *p* = 0.02], whereas prosthetic valve endocarditis was more common in the ST group [OR: 0.34 (95%CI: 0.13, 0.89), *p* = 0.03]. A history of prior cerebrovascular accident was also more prevalent in the ST group [OR: 0.39 (95%CI: 0.22, 0.70), *p* < 0.01] (Supplementary Figure S15).

Baseline characteristics were otherwise comparable between groups, including age [Mean difference (MD): 1.08 (95%CI: −6.79, 8.96), *p* = 0.79], BMI [MD: 0.20 (95%CI: −1.66, 2.07), *p* = 0.83], HTN [OR: 0.87 (95%CI: 0.37, 2.03), *p* = 0.75], DM [OR: 0.87 (95%CI: 0.50, 1.53), *p* = 0.63], male sex [OR: 1.25 (95%CI: 0.82, 1.91), *p* = 0.30], preoperative dialysis [OR: 0.93 (95%CI: 0.32, 2.84), *p* = 0.93], peripheral vascular disease [OR: 0.45 (95%CI: 0.16, 1.25), *p* = 0.13], LVEF [MD: 0.15 (95%CI: −1.9, 2.24), *p* = 0.89], history of prior cardiothoracic surgery [OR: 0.73 (95%CI: 0.13, 4.15), *p* = 0.72] and the presence of active IE [OR: 0.64 (95%CI: 0.19, 2.10), *p* = 0.46].

#### 3.3.2. Intraoperative Details—MIS vs. ST

**Figure 3:**
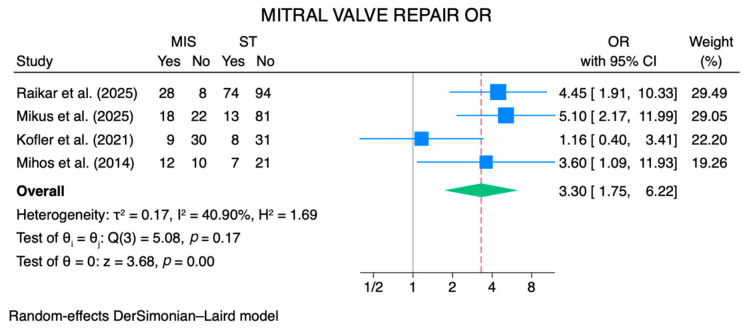
Forest plot illustrating the odds ratio for mitral valve repair in the minimally invasive surgery group compared with the median sternotomy group [25,26,30,34].

MV repair was more frequently performed in the MIS group [OR: 3.30 (95%CI: 1.75–6.22), *p* < 0.01], whereas MV replacement was more common in the ST group [OR: 0.30 (95%CI: 0.16–0.57), *p* < 0.01] (Figure 3). Both CPB time [MD: 23.18 (95%CI: −0.10, 46.47), *p* = 0.05] and aortic cross-clamp time [MD: 15.27 (95%CI: −0.27, 30.80), *p* = 0.05] were comparable between the two groups. Concomitant TV operations were also similar between the two groups [OR: 0.27 (95%CI: 0.02, 4.21), *p* = 0.35].

#### 3.3.3. Postoperative Outcomes—MIS vs. ST

**Figure 4:**
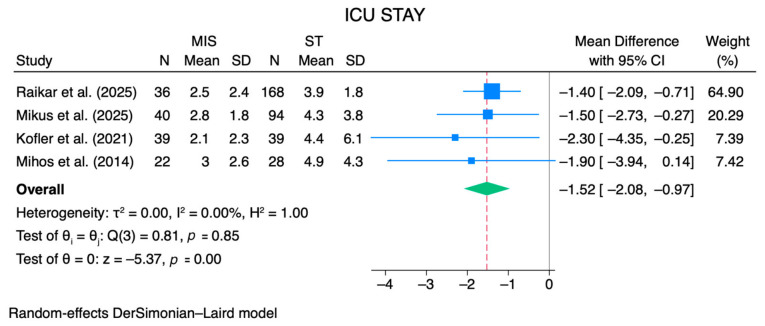
Forest plot illustrating the mean difference (MD) in intensive care unit stay (days) between the minimally invasive surgery and median sternotomy groups [25,26,30,34].

ICU stay was significantly decreased in the MIS group [MD: −1.52 (95%CI: −2.08, −0.97), *p* < 0.01] (Figure 4). Hospital stay was comparable between the two groups [MD: −4.27 (95%CI: −9.06, 0.52), *p* = 0.08].

No difference was observed in the following postoperative complications: arrhythmia [OR: 0.83 (95%CI: 0.37, 1.87), *p* = 0.66], AKI [OR: 0.57 (95%CI: 0.21, 1.54), *p* = 0.27], and stroke [OR: 0.51 (95%CI: 0.11, 2.26), *p* = 0.37]. Pacemaker implantation [OR: 0.62 (95%CI: 0.19, 2.05), *p* = 0.43] and reoperation for bleeding [OR: 0.78 (95%CI: 0.29, 2.13), *p* = 0.63] were also comparable between the two groups.

#### 3.3.4. Mortality OS—MIS vs. ST

**Figure 5:**
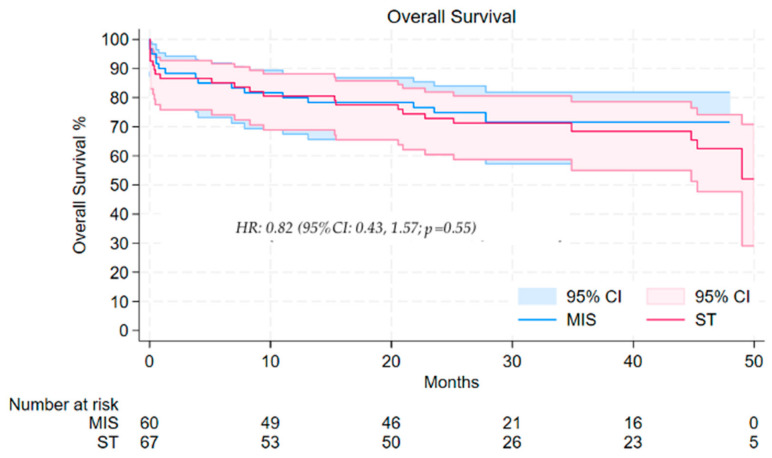
Reconstructed Kaplan–Meier curves comparing overall survival between the minimally invasive surgery and median sternotomy groups.

No differences were observed between the MIS and ST groups in early mortality [OR: 0.64 (95%CI: 0.19, 2.10), *p* = 0.46] or overall mortality [OR: 0.72 (95%CI: 0.34, 1.54), *p* = 0.40]. Long-term survival at 4-year follow-up did not differ significantly between the two groups [Hazard ratio (HR): 0.82 (95%CI: 0.43, 1.57), *p* = 0.55] (Figure 5). The KM curve comparing OS between the MIS and the ST groups was reconstructed using survival data extracted from two studies [30,34].

### 3.4. Risk of Bias Assessment

The quality assessment using the NHLBI scale is summarized in the Supplementary Table S3.

The risk of bias assessment showed an overall moderate to serious risk across the included studies. Most domains, including intervention classification, deviations, missing data, outcome measurement, and selective reporting, were consistently rated as low risk. In contrast, confounding and selection of participants represented the main sources of bias, with confounding rated as moderate to serious and selection bias as low to moderate (Supplementary Figure S16).

### 3.5. Certainty of Evidence Assessment

Due to the inclusion of fewer than 10 studies in the secondary comparative analysis after sensitivity analysis, statistical assessment of publication bias (Egger’s regression) was not performed [39,40,41]. Therefore, publication bias was not formally assessed in the secondary analysis; however, no overt asymmetry was observed qualitatively. The certainty of evidence assessed using the GRADE framework ranged from very low to low across outcomes, with downgrading primarily driven by risk of bias inherent to observational study designs and imprecision due to wide confidence intervals. Outcomes with substantial heterogeneity were further downgraded for inconsistency (Supplementary Table S4).

## 4. Discussion

To our knowledge, this is the first systematic review and meta-analysis of reconstructed time-to-event data to date on minimally invasive surgery (MIS) techniques for mitral valve infective endocarditis (MVIE), incorporating data from around 949 patients across 14 studies. The principal findings are as follows: (1) Overall survival (OS) rates in the MIS group of 86.7% at 1 year, 75.2% at 5 years and 56.2% at 10 years; (2) freedom from infective endocarditis (IE)-related reoperation in the MIS group of 97.5% at 1 year, 95.9% at 5 years and 90.7% at 10 years; and (3) OS was not differ between the MIS and full sternotomy (ST) groups at 4-year follow-up.

First, MIS techniques are highly heterogeneous in terms of surgical approach. In our study, robotic-assisted surgery was performed in approximately 5% of cases. Other techniques include totally endoscopic approaches, right mini-thoracotomy with video assistance, or direct vision techniques. However, the included studies did not clearly report the exact proportion of each of these methods. Hosoba et al. used the Cavitron ultrasound surgical aspirator (CUSA) system, which employs ultrasonic waves and vibrations to fragment and remove calcified material and vegetations, with adjustable intensity to minimize injury to surrounding healthy tissue [29]. However, these different technical variations likely contribute to increased heterogeneity in surgical outcomes across studies. Patient-level data could not be extracted from the included studies, precluding direct comparison of the different approaches and limiting the ability to assess procedure-specific risks associated with each technique.

Mitral valve (MV) repair was performed in 52.5% of patients in our study and was more commonly conducted in the MIS group compared with the ST group. Contemporary series of MVIE report repair rates ranging from 19% to 81%, with the most studies describing rates between 20% and 40% [42,43,44,45,46,47]. The repair rate observed in the MIS cohort in our analysis is notable, particularly considering evidence demonstrating the association of MV repair with improved long-term survival [48]. In contrast, MV replacement has been identified as an independent predictor of mortality [42,49]. The higher prevalence of MV repair in the MIS group may, in part, be explained by the greater proportion of native valve endocarditis in this cohort. At the same time, factors like extensive infection, renal failure and anterior or bileaflet involvement can limit the feasibility of repair, underscoring the importance of careful patient selection to optimize outcomes, particularly in the context of MIS approaches [48,50].

Patient selection remains a critical consideration when choosing between MIS and ST [30]. Reported contraindications to a MIS approach across the included studies include multivalve involvement, extensive disease and involvement of the aortomitral curtain, which may require complex procedures such as the Commando [25,26,28,31,32]. The complexity of the aforementioned pathologies may preclude the use of minimally invasive surgical approaches. However, in the present study, the included comparative analyses excluded such complex cases from both the MIS and ST groups, with recommendations that these patients be managed using conventional surgical techniques [25,26,30]. Preoperative vascular assessment is also essential to evaluate peripheral vascular disease, the incidence of which was similar between the two groups in our study, and ensure the feasibility of peripheral cannulation in MIS patients [27,28]. The presence of shock was considered a contraindication by Barbero et al., whereas in our study, the incidence of shock was 10%, with two studies reporting that this variable was not a contraindication [26,28,29].

Additionally, a history of prior cerebrovascular accident may influence patient selection for MIS. In our meta-analysis, prior cerebrovascular events were more common in the ST group. This may be explained by the fact that previous stroke is a known risk factor for postoperative neurological complications, while MIS techniques have been associated with concerns regarding embolic events [28,51]. Consequently, surgeons may favor a more familiar approach, such as sternotomy, in patients with a history of neurological events. Therefore, establishing clear and individualized surgical selection criteria for MIS in patients with MVIE may help reduce center-based variability in contraindications.

The higher prevalence of prior cerebrovascular accidents and prosthetic valve replacement in the ST group suggests a higher baseline risk profile in this cohort. However, the shorter intensive care unit stay observed in the MIS group is consistent with findings from a previous meta-analysis evaluating minimally invasive approaches in mitral valve surgery [13].

Mean cardiopulmonary bypass (CPB) time and aortic cross-clamp time were 130.6 min and 92.0 min, respectively, in our primary analysis. The mean differences were 23.18 and 15.27, respectively, favoring the ST group, with borderline non-significant differences. MIS techniques have been associated with prolonged surgical times [10,52]. CPB and cross-clamp times in MIS are highly dependent on the learning curve [53]. IE is a complex pathology, and its management using MIS techniques generally reflects prior surgeon experience with minimally invasive approaches in less complex mitral valve disease [30]. Hence, the comparable operative times observed in the included studies may be attributed to substantial surgeon experience with MIS techniques across the reported cohorts. Importantly, both groups remained well below the CPB threshold of >166 min associated with increased mortality in infective endocarditis [54]. Clamping time was above the mortality threshold, but this did not lead to an increase in early or long-term mortality in our analysis [54].

In our analysis, aortic cross-clamping was used in 82.3% of cases, whereas endo-aortic balloon occlusion (EABO) was employed in approximately 18%. Only the study by van der Merwe et al. specifically reported the exclusive use of EABO, highlighting its safety in minimally invasive MV surgery and its potential to reduce embolic events [31,55,56]. A recent meta-analysis further supports the safety of both techniques while emphasizing their distinct advantages. Aortic cross-clamping has been associated with lower rates of conversion to full sternotomy, reduced cerebrovascular events, and shorter intensive care unit stays, whereas EABO has been linked to shorter operative times but a higher risk of aortic dissection. No differences in mortality have been reported between the two approaches [57,58].

A commonly reported advantage of MIS approaches in cardiothoracic surgery is the reduction in both hospital and intensive care unit (ICU) length of stay [52,59,60]. In our primary analysis, the mean hospital and ICU stays were 8.8 and 1.8 days, respectively. In the comparative analysis, a significant reduction was observed only in ICU stay, with a mean decrease of 1.5 days, a finding that may be clinically relevant in terms of postoperative recovery, complication burden, and healthcare cost [61,62,63].

The incidence of postoperative arrhythmia, acute kidney injury, stroke, reoperation for bleeding, and pacemaker implantation was comparable between MIS techniques and the traditional sternotomy approach. In the primary analysis, arrhythmia emerged as the most common postoperative complication. Notably, the observed stroke rate of 1.5% represents a favorable outcome for the minimally invasive cohort when compared with previously reported rates [10,64].

An early mortality rate of 4.2% and overall survival of 86.7%, 75.2% and 56.2% at 1, 5, and 10 years, respectively, represent favorable outcomes. In comparison, a previous systematic review of MIS for MVIE reported an early mortality exceeding 10% and a 1-year survival of 79.3% [65]. The incorporation of reconstructed time-to-event data and meta-analytic techniques in our study, as well as the inclusion of more recent and updated studies, provides a more robust assessment of survival, effectively explaining the differences in outcomes compared to prior reports currently in the literature. Furthermore, the comparable survival observed between minimally invasive and sternotomy groups reinforces these findings.

Freedom from infective endocarditis (IE)-related reoperation was 97.5% at 1 year, 95.9% at 5 years, and 90.7% at 10 years. The studies by Mikus and Hosoba reported no recurrence of IE; consequently, IE-related reoperation was assumed to be 0% over long-term follow-up in these cohorts [26,29]. The study by Barbero et al. provided a Kaplan–Meier curve distinguishing IE-related from non-IE-related reoperations, from which data on IE-related reoperation were extracted and incorporated into the overall reconstructed survival curve [28]. In the study by Kofler et al., no reoperations of any cause were observed on the Kaplan–Meier curve [30]. Finally, the study by Folkmann et al. reported discrete events of IE-related reoperation [32].

This systematic review and meta-analysis of reconstructed time-to-event data synthesizes the available evidence on MIS in MVIE. To our knowledge, this is the first study to apply meta-analytic techniques combined with reconstructed patient-level survival data to evaluate minimally invasive approaches in MVIE, integrating outcomes from approximately 950 patients across published observational studies and providing long-term outcomes. This approach introduces a novel methodological framework for investigating this topic. Previous meta-analyses have examined minimally invasive mitral valve surgery more broadly but have not specifically focused on MVIE [13,58,59]. Thus, the existing evidence on the application of these techniques is limited to observational studies, a gap that supports the rationale for conducting a meta-analysis. However, this approach must be interpreted considering the low certainty of the underlying evidence base. In addition, our study provides a comparative evaluation with sternotomy approaches, suggesting that minimally invasive techniques may offer favorable outcomes when performed by experienced surgeons in appropriately selected patients. Despite these findings, most current guidelines do not specifically address minimally invasive approaches for the management of mitral valve infective endocarditis and continue to favor conventional techniques [6,7,66,67,68]. This evidence gap has been previously acknowledged by several observational studies, including those incorporated in the present analysis [25,26]. By systematically synthesizing the existing literature, our study provides a comprehensive overview of current evidence and may help inform future guideline development, probably by supporting the design of future studies that focus more precisely on individual MIS techniques, which will provide individual patient data and guide the design of more robust comparative investigations in this field.

### Strengths and Limitations

Methodological strengths of this study include: (i) comprehensive literature search using systematic methodology, (ii) detailed reconstruction of time-to-event data at the patient level, (iii) the inclusion of all eligible studies to provide pooled estimates for the MIS cohort alongside a comparative analysis with conventional techniques, (v) novelty, as this is the first systematic review and IPD meta-analysis in the literature to evaluate and summarize the outcomes following MIS in MVIE while simultaneously providing a comparative arm with conventional approaches and (vi) meta-regression performing.

Several limitations should be acknowledged. First, the retrospective nature of all the included studies introduces the potential for selection and confounding bias. More specifically, prosthetic valves and history of cerebrovascular accidents were more prevalent in the ST group, suggesting a higher risk profile in this cohort. Second, the secondary analysis of overall survival was based on reconstructed patient-level data from only two studies, precluding the performance of a two-stage meta-analysis [30,34]. Third, insufficient data were available to investigate the causes of mortality in the primary analysis or to adequately characterize early and late reoperation rates. Similarly, comparisons between minimally invasive and sternotomy groups regarding red blood cell transfusion and intraoperative blood loss were not feasible due to limited reporting. Additionally, comparisons among different minimally invasive techniques were not possible. Moreover, the comparative arm includes only four studies, and the overall certainty of evidence in our study is low to very low. Finally, meta-regression to explore heterogeneity in comparative analysis could not be performed, as fewer than ten comparative studies were available. Collectively, these limitations underscore the need for well-designed prospective and comparative studies to provide more robust evidence in this field.

## 5. Conclusions

The results of this systematic review and meta-analysis of reconstructed time-to-event data indicate that minimally invasive techniques for infective mitral endocarditis are associated with favorable long-term outcomes. Pooled overall survival rates were 86.7%, 75.2%, and 56.2% at 1, 5, and 10 years, respectively, while freedom from infective endocarditis-related reoperation remained high at 97.5%, 95.9%, and 90.7% over the same intervals. Overall survival was not different to conventional techniques at 4-year follow-up. In addition, the intensive care unit stay was significantly shorter in the minimally invasive cohort. Collectively, these findings suggest that minimally invasive surgery may be considered as a feasible and potentially effective alternative for the management of mitral valve infective endocarditis. However, these findings are derived from retrospective data, which inherently limits the strength of the evidence and reduces overall certainty.

## Figures and Tables

**Table 1:** Study characteristics.

Author	Year of Publication	Journal	Center(s)	Country	Design	MIS|ST
Raikar et al. [25]	2025	The Annals of Thoracic Surgery	West Virginia University, Morgantown, West Virginia	USA	Retrospective cohort	36|168
Mikus et al. [26]	2025	Diseases	Maria Cecilia Hospital, GVM Care & Research, Cotignola	Italy	Retrospective cohort	40|94
Franz et al. [27]	2024	Journal of Clinical Medicine	Hannover Medical School, Hannover	Germany	Retrospective cohort	75|NA
Barbero et al. [28]	2023	Medicina	Molinette Hospital, Turin	Italy	Case series	92|NA
Hosoba et al. [29]	2022	Interactive CardioVascular and Thoracic Surgery	Japanese Red Cross Aichi Medical Center Nagoya Daiichi Hospital, Nagoya	Japan	Case series	10|NA
Kofler et al. [30]	2021	European Journal of Cardio-Thoracic Surgery	German Heart Center Berlin, Berlin	Germany	PSM cohort	39|39
van der Merwe et al. [31]	2018	Interactive CardioVascular and Thoracic Surgery	OLV Clinic, Aalst	Belgium	Case series	60|NA
Folkman et al. [32]	2018	The Thoracic and Cardiovascular Surgeon	Heart Center Leipzig, Leipzig	Germany	Case series	92|NA
Glauber et al. [33]	2015	Journal of Cardiothoracic Surgery	Fondazione Toscana G. Monasterio, Via Aurelia Sud, Massa	Italy	Retrospective cohort	80|NA
Mihos et al. [34]	2014	Journal of Heart Valve Disease	Mount Sinai Heart Institute, Miami Beach, Florida	USA	Retrospective cohort	22|28
Chi et al. [35]	2014	Journal of Thoracic Disease	National Taiwan University Hospital and National Taiwan University College of Medicine, Taipei	Taiwan	Case series	12|NA
Jung et al. [36]	2011	European Journal of Cardio-Thoracic Surgery	Asan Medical Center, College of Medicine, University of Ulsan, Seoul	South Korea	Retrospective cohort	24|NA
Martin et al. [37]	2006	Innovations (Philadelphia)	Duke University Medical Center, Durham, North Carolina	USA	Case series	21|NA
Gaudiani et al. [38]	2004	The Heart Surgery Forum	Sequoia Hospital, Redwood City, California	USA	Retrospective cohort	17|NA

NA: Not applicable, PSM: Propensity score matching.

**Table 2:** Characteristics of the diseased mitral valve for the minimally invasive surgery cohort.

Author	*n* (%)						
	Native Valve IE	Prosthetic Valve IE	MR	Vegetations	Abscess	Leaflet Perforation	Anterior Leaflet Involvement
Raikar et al. [25]	NR						
Mikus et al. [26]	35 (87.5)	5 (12.5)		38 (95.0)	1 (2.5)	7 (17.5)	NR
Franz et al. [27]	NR		4 (5.3)			26 (34.7)	
Barbero et al. [28]	80 (87.0)	12 (13.0)	NR				
Hosoba et al. [29]	10 (100)	0 (0.0)	4 (40.0)	10 (100)	NR	1 (10.0)	9 (90.0)
Kofler et al. [30]	39 (100)	0 (0.0)	NR				
van der Merwe et al. [31]	30 (50.0)	30 (50.0)					
Folkman et al. [32]	NR				9 (9.8)	NR	
Glauber et al. [33]							
Mihos et al. [34]	22 (100)	0 (0.0)	NR	12 (54.5)	2 (9.1)	2 (9.1)	8 (36.4)
Chi et al. [35]	12 (100)	0 (0.0)	12 (100)	12 (100)	NR		3 (25.0)
Jung et al. [36]	NR						
Martin et al. [37]							
Gaudiani et al. [38]							

IE: Infective endocarditis, MR: Mitral regurgitation, NR: Not reported.

**Table 3:** Intraoperative characteristics for the minimally invasive surgery cohort.

Author	*n* (%)				CPB Time (SD)	Cross-Clamp Time (SD)	*n* (%)		
	Robotic-Assisted	MV Repair	MV Replacement	TV Surgery			Transthoracic Clamping	EABO	Fibrillatory Arrest
Raikar et al. [25]	32 (88.9)	28 (77.8)	8 (22.2)	0 (0.0)	176.3 ± 62.5	125.9 ± 55.6	NR		
Mikus et al. [26]	0 (0.0)	18 (45.0)	22(55.0)	NR	105.0 ± 33.1	83.5 ± 30.0			
Franz et al. [27]	0 (0.0)	33 (44.0)	42 (56.0)	4 (5.3)	144.0 ± 49.9	80.6 ± 32.5			
Barbero et al. [28]	0 (0.0)	16 (17.4)	76 (82.6)	5 (5.4)	143.0 ± 34.0	105.0 ± 27.0	51 (55.4)	40 (43.5)	1 (1.1)
Hosoba et al. [29]	0 (0.0)	10 (100)	0 (0.0)	NR	124.0 ± 46.0	90.0 ± 38.0	10 (100)	0 (0.0)	0 (0.0)
Kofler et al. [30]	0 (0.0)	9(23.1)	30 (76.9)	0 (0.0)	104.2 ± 46.9	69.7 ± 27.7	NR		
van der Merwe et al. [31]	0 (0.0)	15 (25.0)	45 (75.0)	7 (11.7)	NR		0 (0.0)	60 (100)	0 (0.0)
Folkman et al. [32]	0 (0.0)	23 (25.0)	69 (75.0)	5 (5.4)	113.0	88.0	92 (100)	0 (0.0)	0 (0.0)
Glauber et al. [33]	0 (0.0)	46 (57.5)	34 (42.5)	NR					
Mihos et al. [34]	0 (0.0)	12 (54.5)	10 (45.5)	NR	132.0 ± 44.0	95.0 ± 27.0	22 (100)	0 (0.0)	0 (0.0)
Chi et al. [35]	12 (100)	12 (100)	0 (0.0)		124.0	89.0	12 (100)	0 (0.0)	0 (0.0)
Jung et al. [36]	0 (0.0)	21 (87.5)	3 (12.5)	NR					
Martin et al. [37]	NR	10 (47.5)	11 (52.4)						
Gaudiani et al. [38]	0 (0.0)	5 (29.4)	12 (70.6)						

CPB: Cardiopulmonary bypass, EABO: Endo-aortic balloon occlusion, MV: Mitral valve, NR: Not reported, SD: Standard deviation, TV: tricuspid valve.

## Data Availability

No new data were created or analyzed in this study. Data sharing is not applicable to this article.

## Supplementary Materials

The following supporting information can be downloaded at: https://www.mdpi.com/article/10.3390/jpm16070350/s1, Figure S1: Original (left) and reconstructed (right) Kaplan–Meier curves for overall survival from the study by Barbero et al.; Figure S2: Reconstructed Kaplan–Meier curve for overall survival from the study by Hosoba et al.; Figure S3: Original (left) and reconstructed (right) Kaplan–Meier curves for overall survival from the study by Kofler et al.; Figure S4: Original (left) and reconstructed (right) Kaplan–Meier curves for overall survival from the study by Folkmann et al.; Figure S5: Original (left) and reconstructed (right) Kaplan–Meier curves for overall survival from the study by Mihos et al.; Figure S6: Reconstructed Kaplan–Meier curve for overall survival from the study by Chi et al.; Figure S7: Original (left) and reconstructed (right) Kaplan–Meier curves for freedom from infective endocarditis from the study by Mikus et al.; Figure S8: Original (left) and reconstructed (right) Kaplan–Meier curves for freedom from reoperation from the study by Barbero et al.; Figure S9: Reconstructed Kaplan–Meier curve for freedom from recurrence from the study by Hosoba et al.; Figure S10: Original (left) and reconstructed (right) Kaplan–Meier curves for freedom from reoperation from the study by Kofler et al.; Figure S11: Original (left) and reconstructed (right) Kaplan–Meier curves for freedom from infective endocarditis-related reoperation from the study by Folkmann et al.; Figure S12: Funnel plot for the logit proportion of mitral valve replacement; Figure S13: Meta-regression to explore the effect of year of publication in the logit proportion of mitral valve replacement; Figure S14: Meta-regression to explore the raw proportion of robotic-assisted surgery in the logit proportion of mitral valve replacement; Figure S15: Forest plot illustrating the odds ratio for a history of cerebrovascular accident between the minimally invasive and median sternotomy groups; Figure S16: Traffic light diagram presenting the ROBINS-I method for risk of bias assessment in comparative retrospective studies; Table S1: Baseline characteristics for the minimally invasive surgery cohort; Table S2: Postoperative outcomes for the minimally invasive surgery cohort; Table S3: Quality assessment of included case series using the National Heart, Lung, and Blood Institute (NHLBI) scale; Table S4: Certainty of Evidence Assessment of the primary outcomes using GRADE; Table S5: PRISMA Checklist.

## Abbreviations

The following abbreviations are used in this manuscript:

AKI	Acute Kidney Injury
BMI	Body Mass Index
COPD	Chronic Obstructive Pulmonary Disease
CPB	Cardiopulmonary Bypass
DM	Diabetes Mellitus
EABO	Endo-aortic Balloon Occlusion
ECMO	Extracorporeal Membrane Oxygenation
HR	Hazard Ratio
HTN	Hypertension
ICU	Intensive Care Unit
IE	Infective Endocarditis
IPD	Individual Patient Data
KM	Kaplan–Meier
LVEF	Left Ventricular Ejection Fraction
MIS	Minimally Invasive Surgery
MV	Mitral Valve
MVIE	Mitral Valve Infective Endocarditis
NHLBI	National Heart, Lung, and Blood Institute
OS	Overall Survival
PICO	Population, Intervention, Comparison, Outcome
PRISMA	Preferred Reporting Items for Systematic Reviews and Meta-Analyses
ROBINS-I	Risk Of Bias In Non-randomized Studies of Interventions
SD	Standard Deviation
ST	Sternotomy
TV	Tricuspid Valve
