# Supplementary material for: Minimally Invasive Surgery for Mitral Valve Endocarditis: A Systematic Review and Meta-Analysis of Reconstructed Time-to-Event Data
Source: J Pers Med. 2026 Jun 29;16(7):350. doi: 10.3390/jpm16070350 (PMC13412392; doi:10.3390/jpm16070350)
Supplement: Supplementary file 1 [file jpm-16-00350-s001.zip › jpm-4318065-supplementary.pdf]

Supplementary Figure S1. Original (left) and reconstructed (right) Kaplan–Meier curves for overall survival from the study by Barbero et al [28].

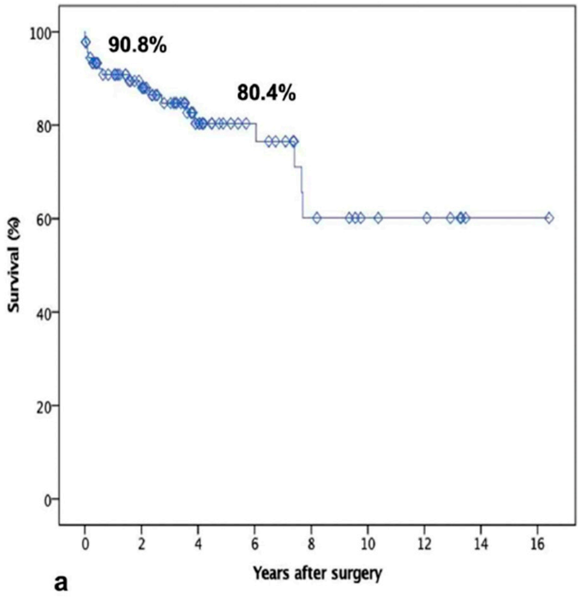

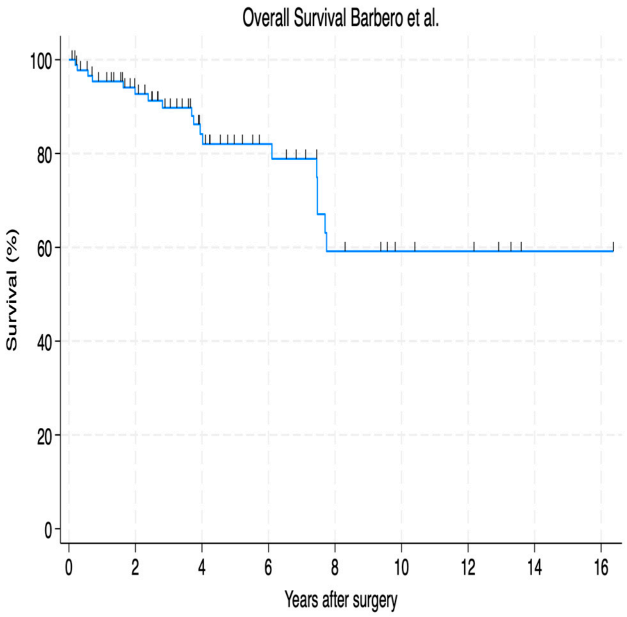

Supplementary Figure S2. Reconstructed Kaplan–Meier curve for overall survival from the study by Hosoba et al [29].

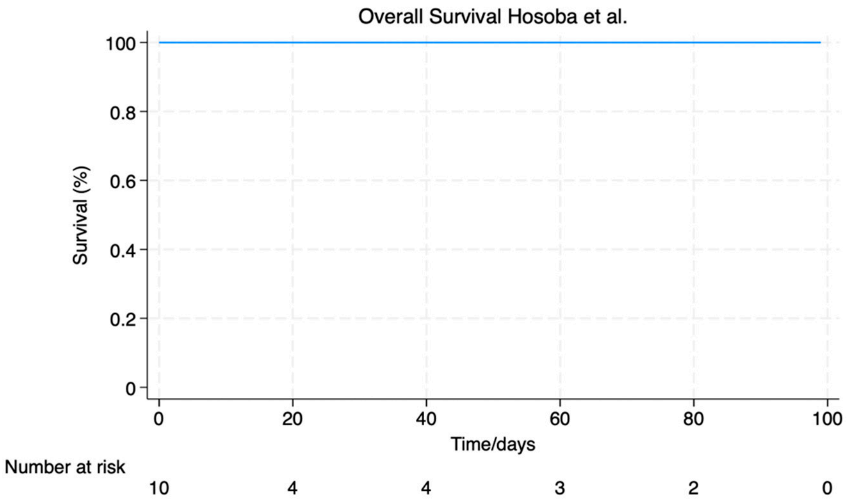

Supplementary Figure S3. Original (left) and reconstructed (right) Kaplan–Meier curves for overall survival from the study by Kofler et al [30].

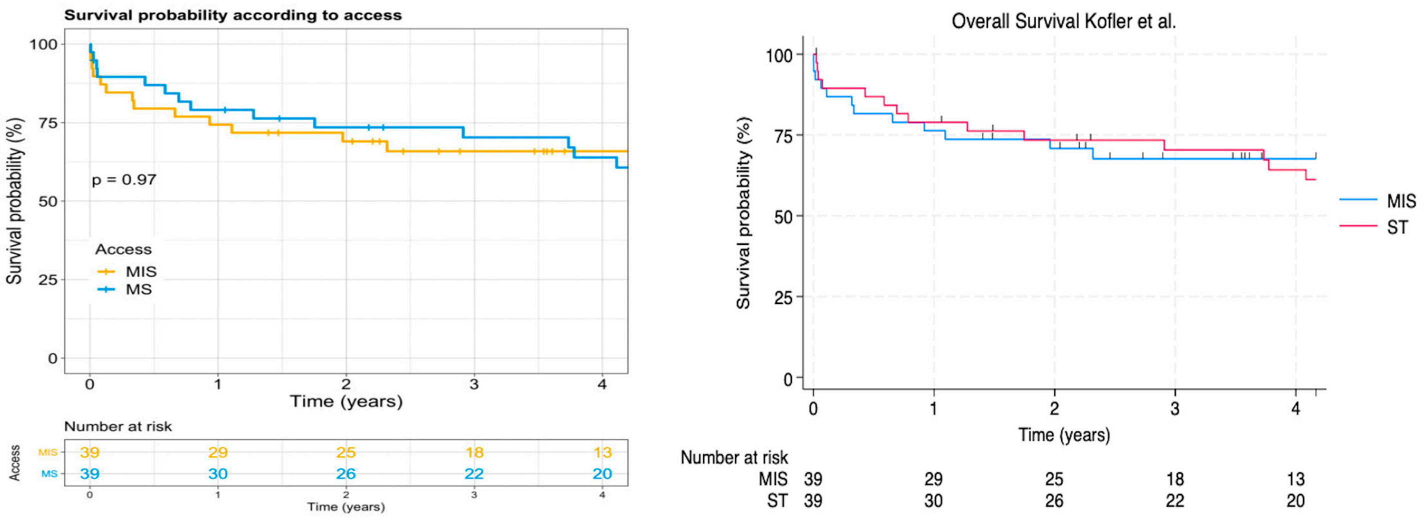

Supplementary Figure S4. Original (left) and reconstructed (right) Kaplan–Meier curves for overall survival from the study by Folkmann et al [32].

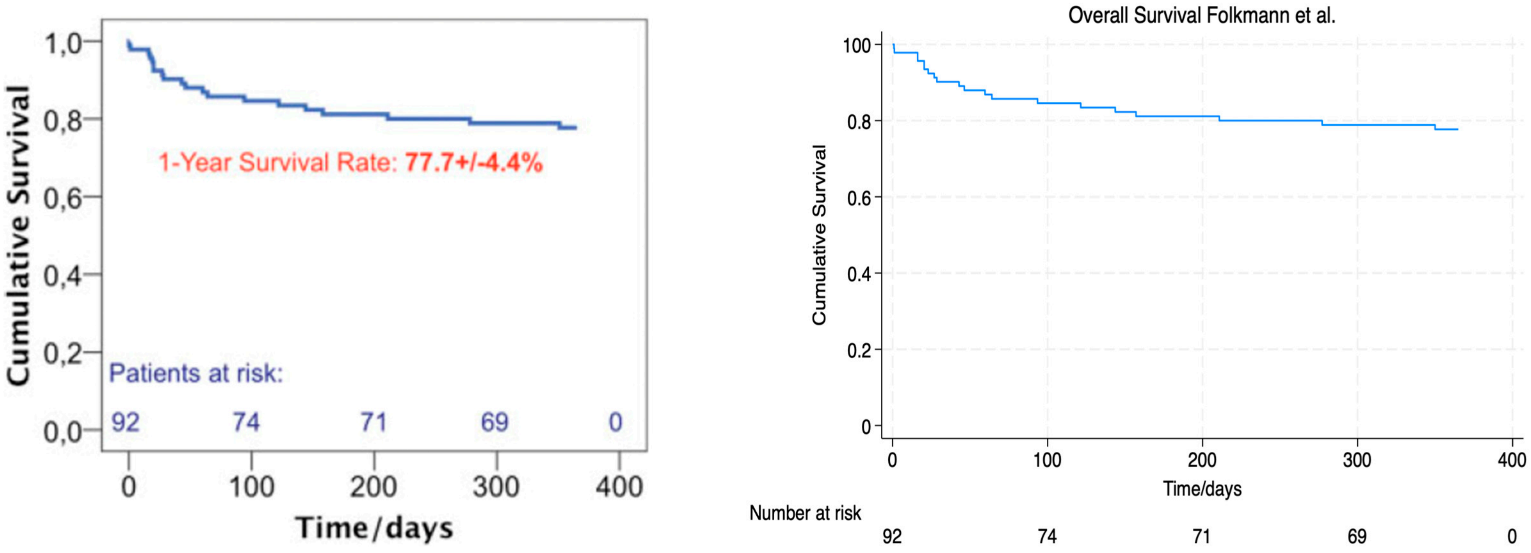

Supplementary Figure S5. Original (left) and reconstructed (right) Kaplan–Meier curves for overall survival from the study by Mihos et al [34].

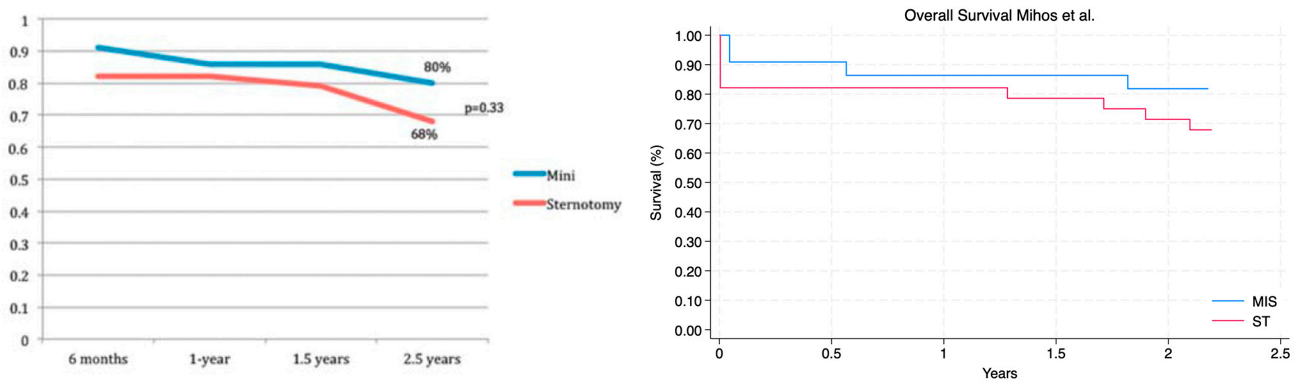

Supplementary Figure S6. Reconstructed Kaplan–Meier curve for overall survival from the study by Chi et al [35].

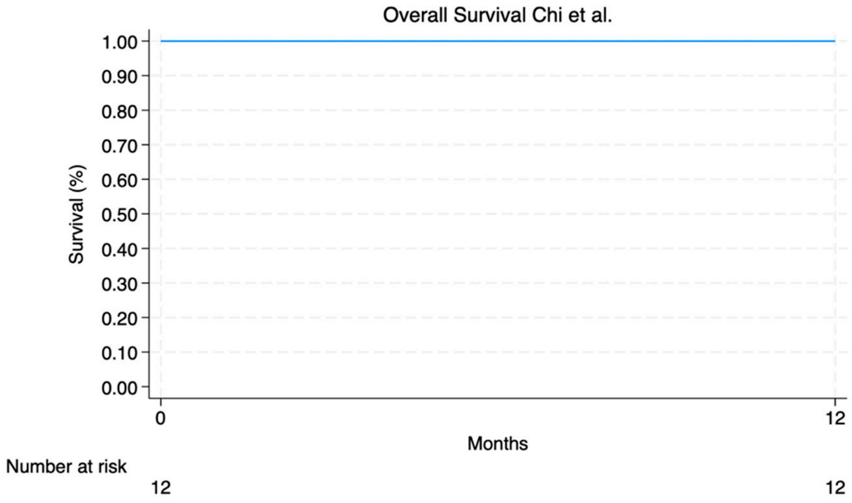

Supplementary Figure S7. Original (left) and reconstructed (right) Kaplan–Meier curves for freedom from infective endocarditis from the study by Mikus et al [26].

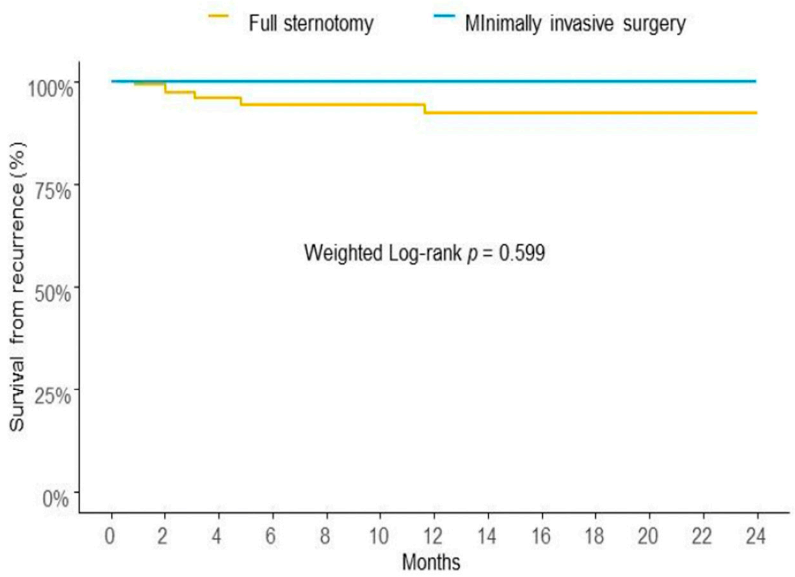

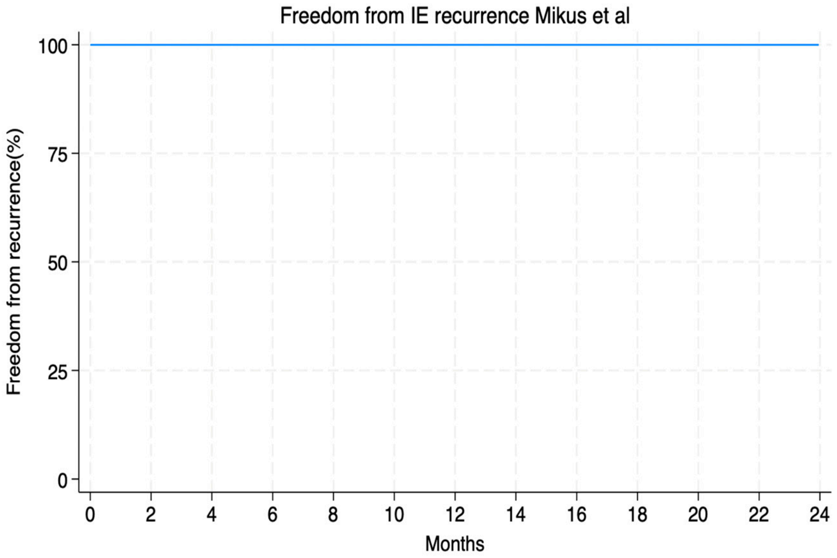

Supplementary Figure S8. Original (left) and reconstructed (right) Kaplan–Meier curves for freedom from reoperation from the study by Barbero et al [28].

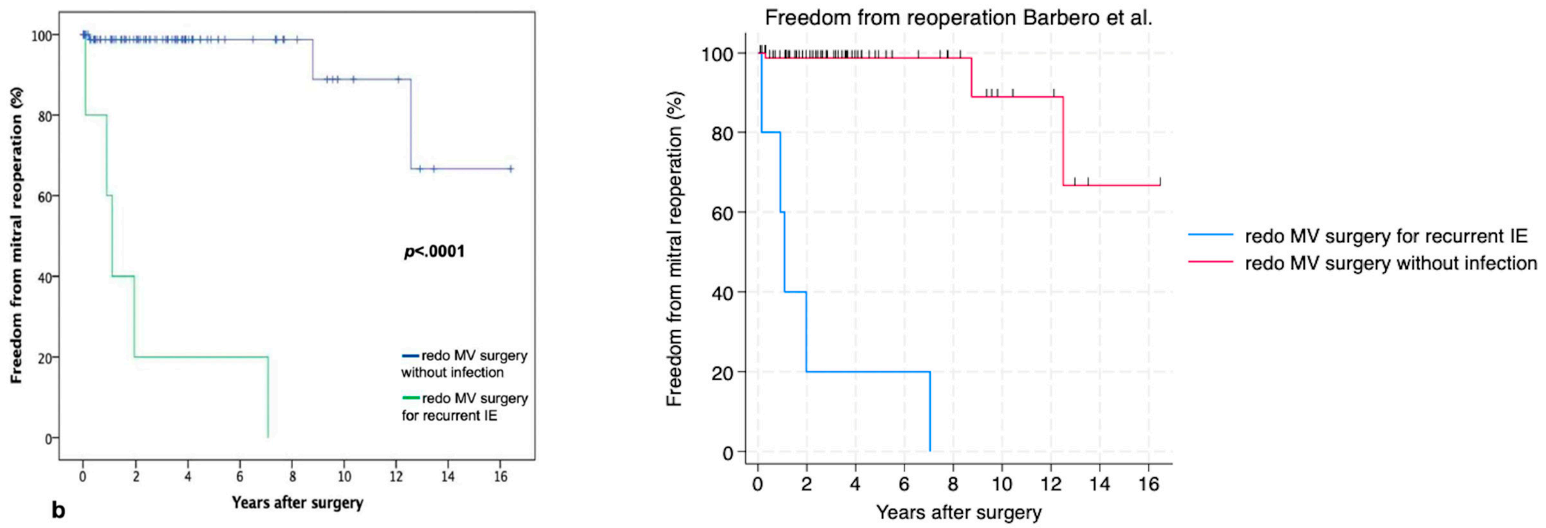

Supplementary Figure S9. Reconstructed Kaplan–Meier curve for freedom from recurrence from the study by Hosoba et al [29].

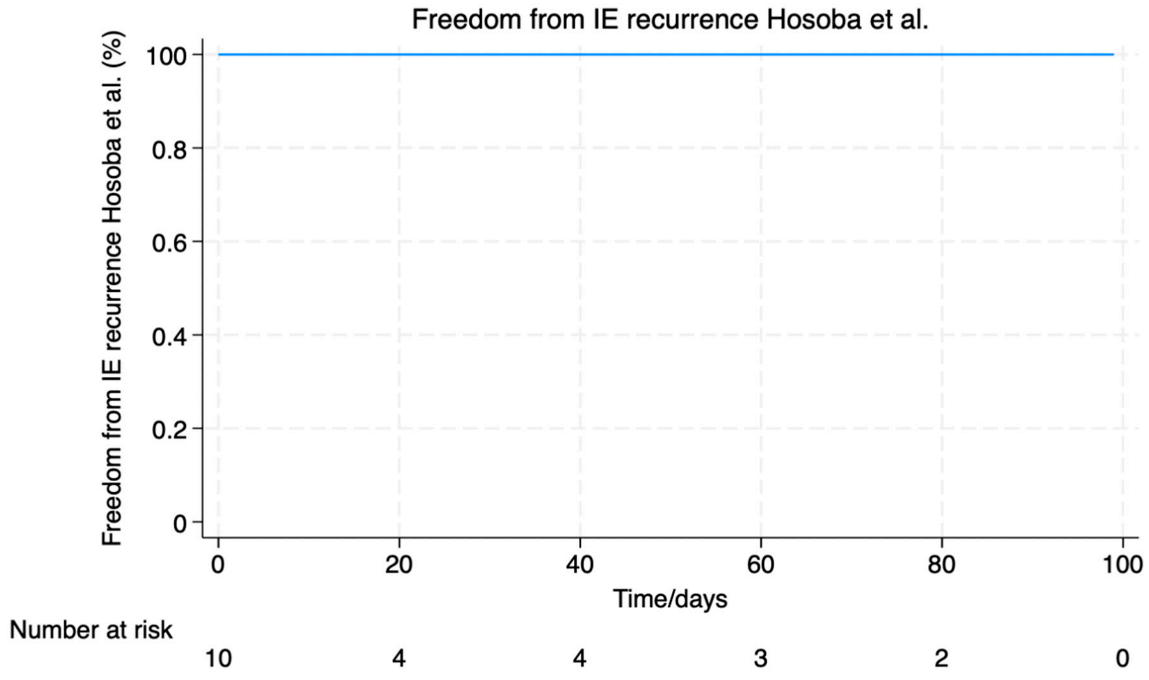

Supplementary Figure S10. Original (left) and reconstructed (right) Kaplan–Meier curves for freedom from reoperation from the study by Kofler et al [30].

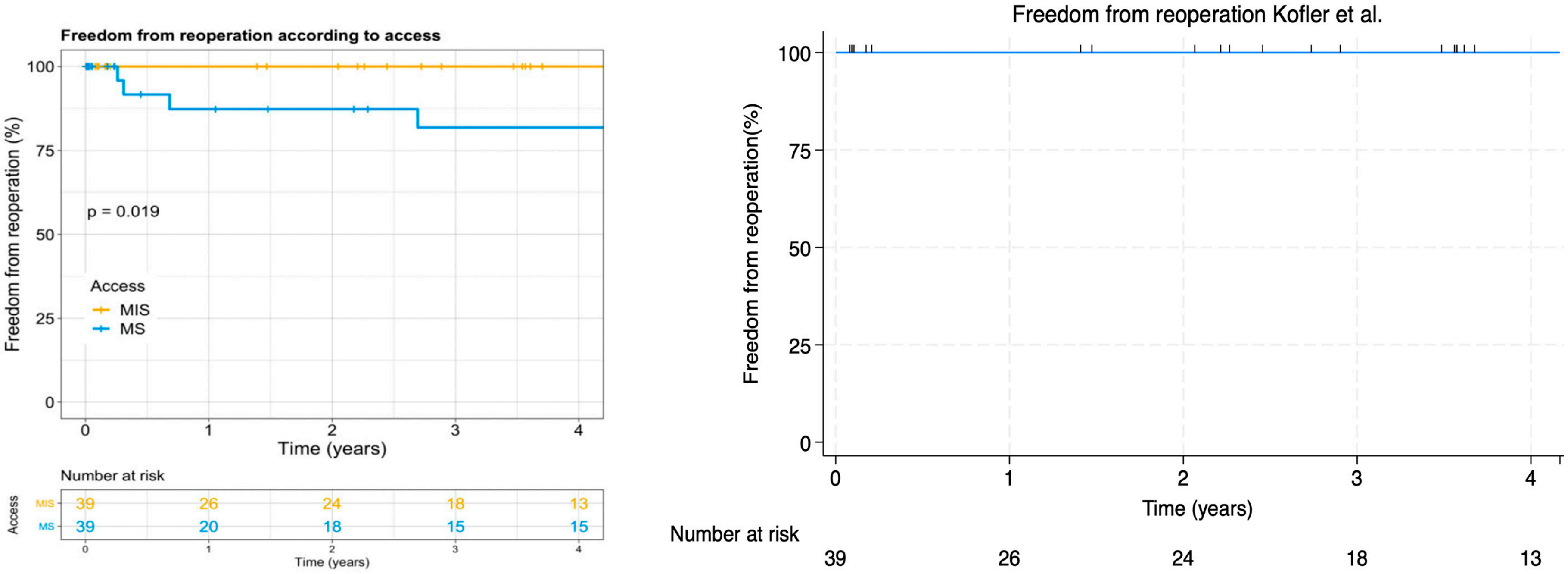

Supplementary Figure S11. Original (left) and reconstructed (right) Kaplan–Meier curves for freedom from infective endocarditis-related reoperation from the study by Folkmann et al [32].

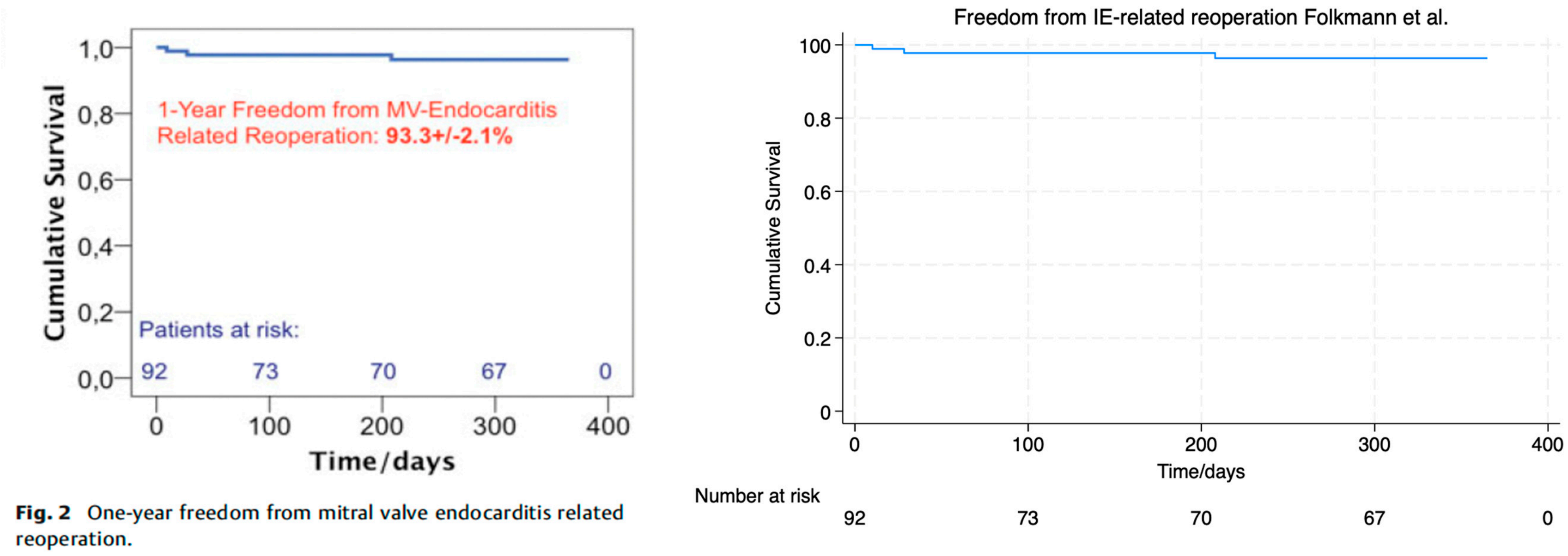

Supplementary Figure S12. Funnel plot for the logit proportion of mitral valve replacement.

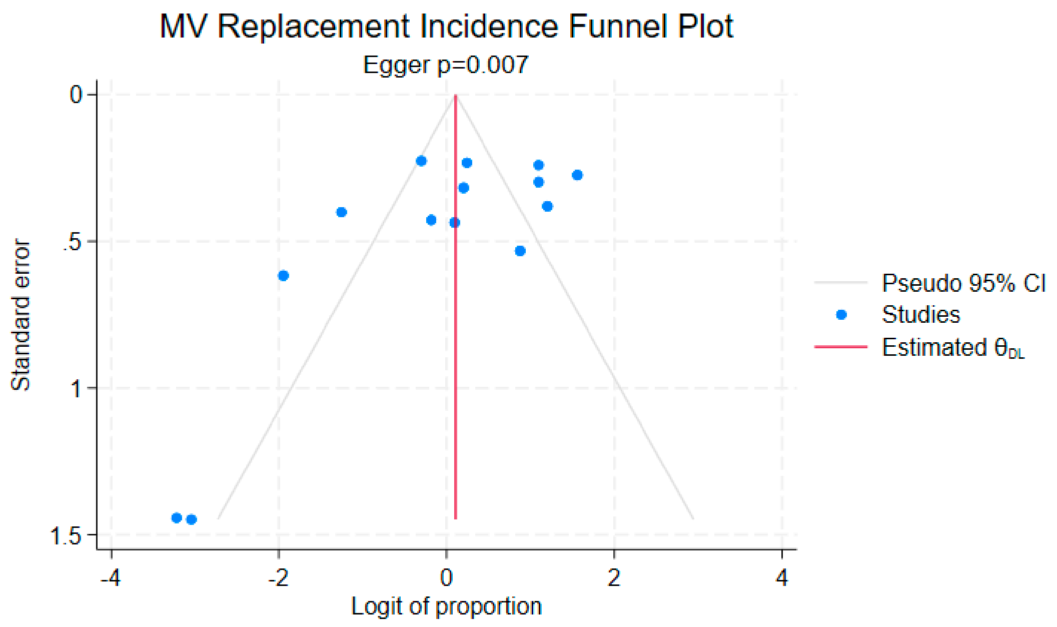

Supplementary Figure S13. Meta-regression to explore the effect of year of publication in the logit proportion of mitral valve replacement.

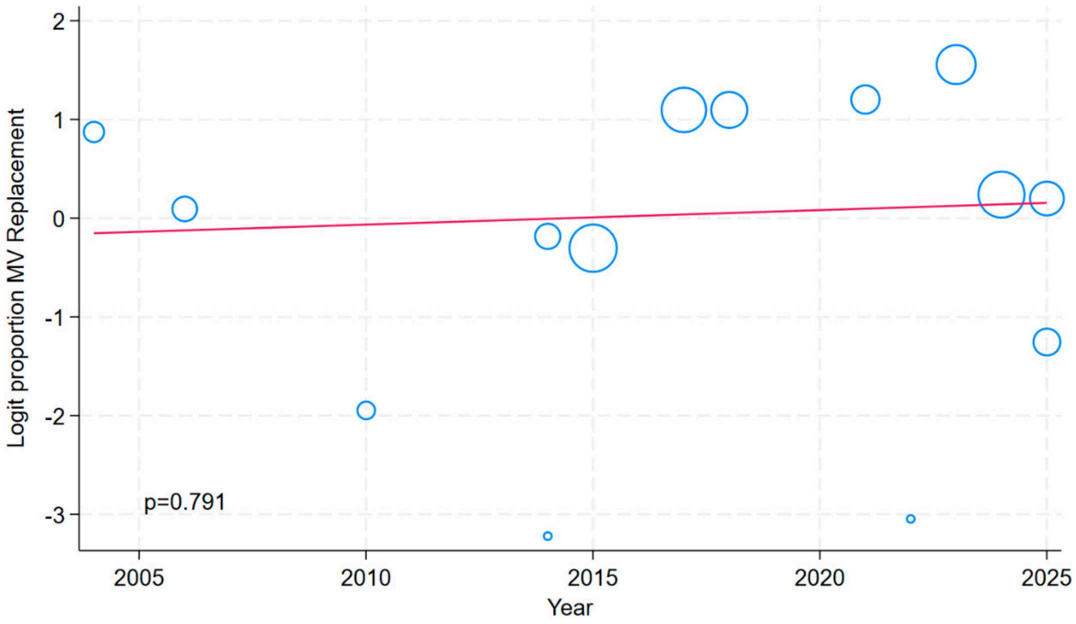

Supplementary Figure S14. Meta-regression to explore the raw proportion of robotic-assisted surgery in the logit proportion of mitral valve replacement.

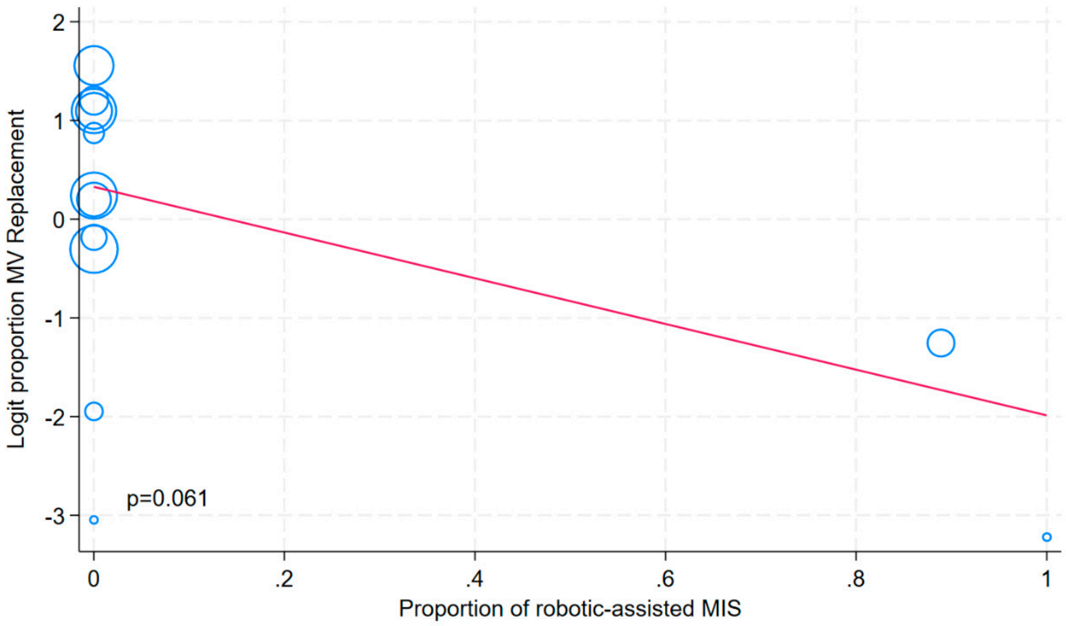

Supplementary Figure S15. Forest plot illustrating the odds ratio for a history of cerebrovascular accident between the minimally invasive and median sternotomy groups [25,26,34].

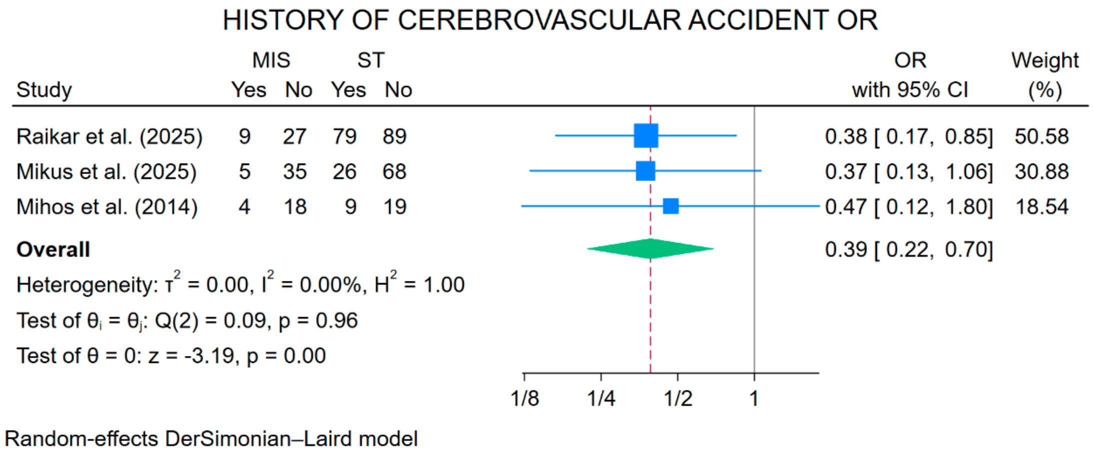

Supplementary Figure S16. Traffic light diagram presenting the ROBINS-I method for risk of bias assessment in comparative retrospective studies [25,26,27,30,34,36,38].

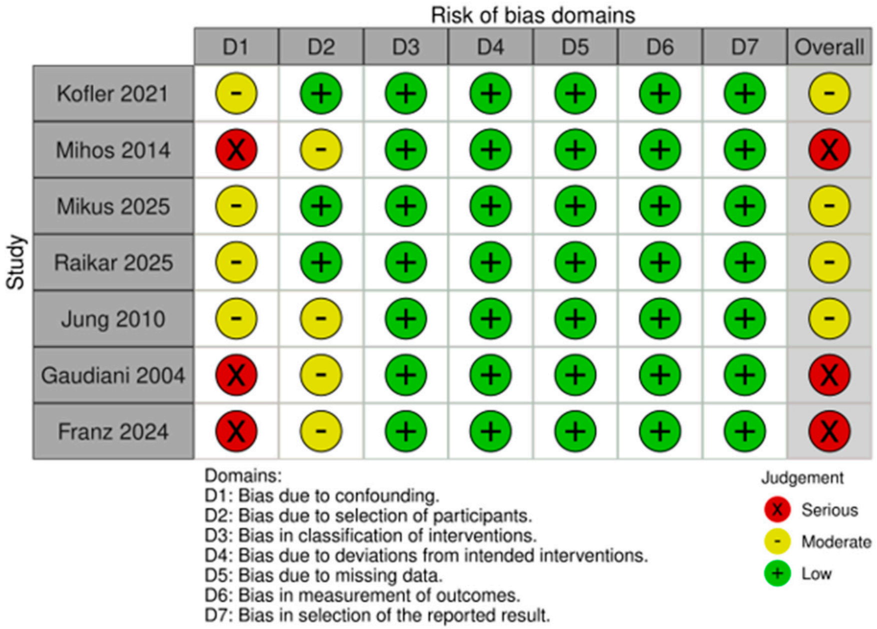

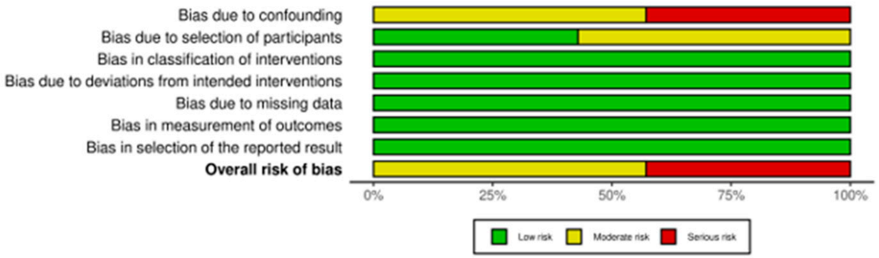

Supplementary Table S1. Baseline characteristics for the minimally invasive surgery cohort

Author	Age (SD)	n (%)							
		Male	COPD	HTN	DM	Hx of CT	Hx of CVA	PVD	Active IE
Raikar et al. (2025) [25]	54.3 ± 13.1	21 (58.3)		23 (63.9)	9 (25.0)	3 (8.3)	9 (25.0)	1 (2.8)	19 (52.8)
Mikus et al. (2025) [26]	59.9 ± 18.4	22 (55.0)	4 (10.0)	19 (47.5)	9 (22.5)		5 (12.5)	2 (5.0)	27 (67.5)
Franz et al. (2024). [27]	59.6 ± 14.4	49 (65.3)	7 (9.3)	37 (49.3)	2 (2.7)	11 (14.7)	40 (53.3)	2 (2.0)	75 (100)
Barbero et al. (2023) [28]	61.1 ± 12.7	58 (63.0)	7 (7.6)	44 (47.8)	18 (19.6)	26 (28.3)	13 (14.1)	2 (2.2)	NR
Hosoba et al. (2022)[29]	55.0 ± 15.0	5 (50.0)			NR				10 (100)
Kofler et al. (2021)[30]	56.4 ± 17.0	23 (59.0)		22 (56.4)	6 (15.4)	2 (5.1)		NR	
van der Merwe et al. (2018).[31]					NR				
Folkman et al. (2017)[32]	60.9 ± 15.3	59 (64.1)			31 (33.7)	14 (15.2)			
Glauber et al. (2015)[33]					NR				
Mihos et al. (2014)[34]	65.0 ± 12.0	17 (77.3)	9 (40.9)	18 (81.8)	8 (36.4)	4 (18.2)	4 (18.2)	2 (9.1)	13 (59.1)
Chi et al. (2014). [35]	43				NR				

Jung et al. (2010) [36]	NR
Martin et al. (2006) [37]	
Gaudiani et al. (2004)[38]	

CT: Cardiothoracic, COPD: Chronic obsructive disease, CVA: Cerebrovascular accident, DM: Diabetes mellitus, HTN: Hypertension, Hx: History, IE: Infective endocarditis, NR: Not reported, PVD: Peripheral vascular disease, SD: Standard deviation

Supplementary Table S2. Postoperative outcomes for the minimally invasive surgery cohort

Author	Hospital stay (SD)	ICU stay (SD)	Complications n (%)						n (%)		
			Ventilation time	Arrhythmia	Stroke	Sepsis	AKD	PM implantation	Reintubation	Reoperation for bleeding	Early mortality
Raikar et al. (2025)[25]	12.5 ± 15.4	2.5 ± 2.4	NR	9 (25.0)	0 (0.0)	NR	1 (2.8)		NR		0 (0.0)
Mikus et al. (2025)[26]	7.3 ± 3.8	2.8 ± 1.8	7.3 ± 5.4	8 (20.0)	1 (2.5)	1 (2.5)	4 (10.0)	1 (2.5)	NR	2 (5.0)	NR
Franz et al. (2024)[27]	NR	1.0 ± 1.5	13.1 ± 9.1	3 (4.0)	2 (2.7)	1 (1.3)	4 (5.3)	1 (1.3)	NR		4 (5.3)
Barbero et al. (2023)[28]	8.0 ± 3.8	1.3 ± 0.7	NR		1 (1.1)		5 (5.4)	3 (3.3)	3 (3.3)		4 (4.4)
Hosoba et al. (2022)[29]			NR		0 (0.0)			NR			0 (0.0)
Kofler et al. (2021)[30]	NR	2.1 ± 2.3	13.3 ± 10.3	NR	1 (2.6)	NR		1 (2.6)	2 (5.1)	5 (12.8)	4 (10.3)
van der Merwe et al. (2018)[31]			NR		NR			NR			
Folkman et al. (2017)[32]					5 (5.4)	2 (2.2)					9 (9.8)

Glauber et al. (2015)[33]											
Mihos et al. (2014)[34]	9.1 ± 5.5	3.0 ± 2.6	NR	2 (9.1)	0 (0.0)	0 (0.0)	0 (0.0)	3 (13.6)	4 (18.2)	0 (0.0)	1 (4.5)
Chi et al. (2014)[35]			NR		0 (0.0)			NR			0 (0.0)
Jung et al. (2010)[36]						NR					
Martin et al. (2006)[37]											
Gaudiani et al. (2004)[38]											

AKD: Acute kidney injury, ICU: Intensive care unit, NR: Not reported, PM: Pacemaker, SD: Standard deviation

Supplementary Table S3. Quality assessment of included case series using the National Heart, Lung, and Blood Institute (NHLBI) scale.

Author	Q1: Objective clearly stated	Q2: Population clearly described	Q3: Consecutive cases	Q4: Subjects comparable	Q5: Intervention clearly described	Q6: Outcomes clearly defined	Q7: Follow-up adequate	Q8: Statistical methods described	Q9: Results clearly described	Overall Quality
Barbero 2023[28]	Yes	Yes	Yes	Yes	Yes	Yes	Yes	Yes	Yes	Good
Folkmann 2018[32]	Yes	Yes	Yes	Yes	Yes	Yes	Yes	Yes	Yes	Good
van der Merwe 2018[31]	Yes	Yes	Yes	Yes	Yes	Yes	Yes	Yes	Yes	Fair
Glauber 2015[33]	Yes	Yes	Yes	Yes	Yes	Yes	Yes	Yes	Yes	Good
Martin 2006[37]	Yes	Yes	Yes	Yes	Yes	Yes	Yes	Yes	Yes	Fair
Franz 2024[27]	Yes	Yes	Yes	Yes	Yes	Yes	Yes	Yes	Yes	Good
Chi 2014[35]	Yes	Yes	No	No	Yes	Yes	No	No	Yes	Poor

Hosoba 2022[29]	Yes	Yes	No	No	Yes	No	No	No	Yes	Poor

NR: Not reported

Supplementary Table S4: Certainty of Evidence Assessment of the primary outcomes Using GRADE

Outcome	No. of studies	Study design	Risk of bias	Inconsistency	Indirectness	Imprecision	Publication bias	Relative effect (95% CI)	Certainty
CPB time	4	Observational	Serious[a]	Very serious[b]	Not serious	Not serious	Not assessed[d]	MD 23.18 (-0.10,46.47)	⊕◯◯◯ Very low
Cross-clamp time	4	Observational	Serious[a]	Very serious[b]	Not serious	Not serious	Not assessed[d]	MD 15.27 (-0.27,30.80)	⊕◯◯◯ Very low
Early mortality	3	Observational	Serious[a]	Not serious	Not serious	Serious[c]	Not assessed[d]	OR 0.64 (0.19–2.10)	⊕⊕◯◯ Low
Postoperative Stroke	4	Observational	Serious[a]	Not serious	Not serious	Serious[c]	Not assessed[d]	OR 0.51 (0.11–2.26)	⊕⊕◯◯ Low
Postoperative Arrhythmia	3	Observational	Serious[a]	Serious[b]	Not serious	Serious[c]	Not assessed[d]	OR 0.83 (0.37–1.87)	⊕◯◯◯ Very low
Postoperative kidney injury	3	Observational	Serious[a]	Not serious	Not serious	Serious[c]	Not assessed[d]	OR 0.57 (0.21–1.54)	⊕⊕◯◯ Low
Reoperation for bleeding	3	Observational	Serious[a]	Not serious	Not serious	Serious[c]	Not assessed[d]	OR 0.78 (0.29–2.13)	⊕⊕◯◯ Low

Permanent pacemaker	3	Observational	Serious[a]	Not serious	Not serious	Serious[c]	Not assessed[d]	OR 0.62 (0.19–2.05)	⊕⊕◯◯ Low
ICU stay	4	Observational	Serious[a]	Not serious	Not serious	Not serious	Not assessed[d]	MD −1.52 (-2.08,-0.97)	⊕⊕◯◯ Low
Hospital stay	3	Observational	Serious[a]	Very serious	Not serious	Not serious	Not assessed[d]	MD −4.27 (-9.06,0.52)	⊕◯◯◯ Very low

CI: confidence interval; **MD:** mean difference; **OR:** odds ratio; **ICU:** Intensive Care Unit**; CPB:** Cardiopoulmonary Bypass

Explanation:

a. **Risk of bias:** Several included studies were observational with high risk of bias according to methodological assessment tools (e.g., selection bias, confounding).

b. **Inconsistency:** Heterogeneity was observed across studies, likely reflecting differences in study design, patient populations, and perioperative management.

- *Serious* = moderate heterogeneity across studies
- *Very serious* = substantial heterogeneity (I^2 >75%), indicating important variability in effect estimates

c. **Imprecision:** Confidence intervals were wide and crossed the line of no effect or were based on a limited number of events.

d.**Publication bias:** Formal statistical assessment of publication bias was not performed because fewer than 10 studies were available for each outcome.

Supplementary Table S5. PRISMA Checklist

Section and Topic	Item #	Checklist item	Location where item is reported
**TITLE**			
Title	1	Identify the report as a systematic review.	Lines 1-3
**ABSTRACT**			
Abstract	2	See the PRISMA 2020 for Abstracts checklist.	Lines 16-35
**INTRODUCTION**			
Rationale	3	Describe the rationale for the review in the context of existing knowledge.	Lines 39-57
Objectives	4	Provide an explicit statement of the objective(s) or question(s) the review addresses.	Lines 58-64
**METHODS**			
Eligibility criteria	5	Specify the inclusion and exclusion criteria for the review and how studies were grouped for the syntheses.	Lines 71-76
Information sources	6	Specify all databases, registers, websites, organisations, reference lists and other sources searched or consulted to identify studies. Specify the date when each source was last searched or consulted.	Line 90
Search strategy	7	Present the full search strategies for all databases, registers and websites, including any filters and limits used.	Lines 91-93
Selection process	8	Specify the methods used to decide whether a study met the inclusion criteria of the review, including how many reviewers screened each record and each report retrieved, whether they worked independently, and if applicable, details of automation tools used in the process.	Lines 76-91
Data collection process	9	Specify the methods used to collect data from reports, including how many reviewers collected data from each report, whether they worked independently, any processes for obtaining or confirming data from study investigators, and if applicable, details of automation tools used in the process.	Lines 100-102
Data items	10a	List and define all outcomes for which data were sought. Specify whether all results that were compatible with each outcome domain in each study were sought (e.g. for all measures, time points, analyses), and if not, the methods used to decide which results to collect.	Lines 103-123
	10b	List and define all other variables for which data were sought (e.g. participant and intervention characteristics, funding sources). Describe any assumptions made about any missing or unclear information.	Lines 142-153
Study risk of bias assessment	11	Specify the methods used to assess risk of bias in the included studies, including details of the tool(s) used, how many reviewers assessed each study and whether they worked independently, and if applicable, details of automation tools used in the process.	Lines 174-184
Effect measures	12	Specify for each outcome the effect measure(s) (e.g. risk ratio, mean difference) used in the synthesis or presentation of results.	Lines 127-140
Synthesis methods	13a	Describe the processes used to decide which studies were eligible for each synthesis (e.g. tabulating the study intervention characteristics and comparing against the planned groups for each synthesis (item #5)).	Supplementary Figure 1
	13b	Describe any methods required to prepare the data for presentation or synthesis, such as handling of missing summary statistics, or data	Lines 127-129

Section and Topic	Item #	Checklist item	Location where item is reported
		conversions.	
	13c	Describe any methods used to tabulate or visually display results of individual studies and syntheses.	Lines 136-137 & Lines 155-164
	13d	Describe any methods used to synthesize results and provide a rationale for the choice(s). If meta-analysis was performed, describe the model(s), method(s) to identify the presence and extent of statistical heterogeneity, and software package(s) used.	Lines 137-140
	13e	Describe any methods used to explore possible causes of heterogeneity among study results (e.g. subgroup analysis, meta-regression).	Lines 166-172
	13f	Describe any sensitivity analyses conducted to assess robustness of the synthesized results.	Lines 166-172
Reporting bias assessment	14	Describe any methods used to assess risk of bias due to missing results in a synthesis (arising from reporting biases).	Lines 175-184
Certainty assessment	15	Describe any methods used to assess certainty (or confidence) in the body of evidence for an outcome.	Lines 187-198
**RESULTS**			
Study selection	16a	Describe the results of the search and selection process, from the number of records identified in the search to the number of studies included in the review, ideally using a flow diagram.	Supplementary Figure 1
	16b	Cite studies that might appear to meet the inclusion criteria, but which were excluded, and explain why they were excluded.	Line 205
Study characteristics	17	Cite each included study and present its characteristics.	Line 206
Risk of bias in studies	18	Present assessments of risk of bias for each included study.	Lines 375-383
Results of individual studies	19	For all outcomes, present, for each study: (a) summary statistics for each group (where appropriate) and (b) an effect estimate and its precision (e.g. confidence/credible interval), ideally using structured tables or plots.	Tables 2-4 & Supplementary tables 1-2
Results of syntheses	20a	For each synthesis, briefly summarise the characteristics and risk of bias among contributing studies.	Supplementary Table 3 & Supplementary Figure 6
	20b	Present results of all statistical syntheses conducted. If meta-analysis was done, present for each the summary estimate and its precision (e.g. confidence/credible interval) and measures of statistical heterogeneity. If comparing groups, describe the direction of the effect.	Lines 219-308
	20c	Present results of all investigations of possible causes of heterogeneity among study results.	Lines 311-369

Section and Topic	Item #	Checklist item	Location where item is reported
	20d	Present results of all sensitivity analyses conducted to assess the robustness of the synthesized results.	Lines 311-369
Reporting biases	21	Present assessments of risk of bias due to missing results (arising from reporting biases) for each synthesis assessed.	Lines 375-383
Certainty of evidence	22	Present assessments of certainty (or confidence) in the body of evidence for each outcome assessed.	Lines 385-394
**DISCUSSION**			
Discussion	23a	Provide a general interpretation of the results in the context of other evidence.	Lines 397-404
	23b	Discuss any limitations of the evidence included in the review.	Lines 505-517
	23c	Discuss any limitations of the review processes used.	Lines 505-517
	23d	Discuss implications of the results for practice, policy, and future research.	Lines 487-495
**OTHER INFORMATION**			
Registration and protocol	24a	Provide registration information for the review, including register name and registration number, or state that the review was not registered.	Line 97
	24b	Indicate where the review protocol can be accessed, or state that a protocol was not prepared.	Line 96-97
	24c	Describe and explain any amendments to information provided at registration or in the protocol.	Not reported
Support	25	Describe sources of financial or non-financial support for the review, and the role of the funders or sponsors in the review.	Line 539
Competing interests	26	Declare any competing interests of review authors.	Line 545
Availability of data, code and other materials	27	Report which of the following are publicly available and where they can be found: template data collection forms; data extracted from included studies; data used for all analyses; analytic code; any other materials used in the review.	Line 542-543
